# Supplementary material for: Reconciling Mining with the Conservation of Cave Biodiversity: A Quantitative Baseline to Help Establish Conservation Priorities
Source: PLoS One. 2016 Dec 20;11(12):e0168348. doi: 10.1371/journal.pone.0168348 (PMC5173368; doi:10.1371/journal.pone.0168348)
Supplement: S1 Dataset — (ZIP) [file pone.0168348.s002.zip › Taxa/Serra Norte/SN_2013/N5S/Lista N5S-04.pdf]

CAVIDADE N5S-0004

Classe	Ordem	Fam/Outros	Gên/Outros	Espécie	S-U	U-S	Zon.
Arachnida	Acari	Rhagidiidae		sp.20	X		P
Arachnida	Acari	Trombidiformes		sp.49	X		P
Arachnida	Acari			jovem	X	X	
Arachnida	Amblypygi	Charinidae	*Charinus*	sp.1	2		P
Arachnida	Amblypygi	Phrynidae	*Heterophrynus*	*longicornis*	1		P
Arachnida	Araneae	Araneidae		jovem	X		P
Arachnida	Araneae	Caponiidae		sp.1	X	X	P/A
Arachnida	Araneae	Corinnidae		jovem	1		A
Arachnida	Araneae	Ctenidae	*Ctenus*	sp.2	2		P
Arachnida	Araneae	Ochyroceratidae	*Gen.*n.	sp.1		X	
Arachnida	Araneae	Ochyroceratidae	*Ochyrocera*	sp.3	X		P
Arachnida	Araneae	Ochyroceratidae	*Speocera*	sp.1	X		A
Arachnida	Araneae	Oonopidae	aff. *Triaeris*	sp.2	X		P
Arachnida	Araneae	Oonopidae	aff. *Xyccarph*	sp.1	X		P
Arachnida	Araneae	Pholcidae	*Leptopholcus*	sp.1	X		P
Arachnida	Araneae	Pholcidae	*Mesabolivar*	sp.1	X		E
Arachnida	Araneae	Salticidae		sp.7	X		E
Arachnida	Araneae	Symphytognathidae	*Anapistula*	sp.1	X		E
Arachnida	Araneae	Theridiidae	*Theridion*	sp.1	X	X	E
Arachnida	Araneae	Theridiosomatidae	*Plato*	sp.1	X	X	P
Arachnida	Opiliones	Cosmetidae	*Roquettea*	*singularis*	1		E
Arachnida	Opiliones	Escadabiidae		sp.1	X	X	P/A
Chilopoda	Scolopendromorpha	Scolopocryptopidae	*Newportia*	sp.1	3		E/P
Chilopoda	Scolopendromorpha	Scolopocryptopidae	*Tidops*	sp.1	1		P
Diplopoda	Polydesmida	Fuhrmannodesmidae		jovem		X	
Diplopoda	Polydesmida	Pyrgodesmidae		sp.1	X	X	A
Entognatha	Diplura	Campodeidae		sp.1		X	
Insecta	Blattodea	Blattellidae		sp.1	2		E/A
Insecta	Blattodea	Blattidae		sp.5	3		P
Insecta	Blattodea	Polyphagidae		sp.1	5		P
Insecta	Blattodea			jovem	3	X	P
Insecta	Coleoptera	Chrysomelidae		sp.4	X		P
Insecta	Coleoptera	Elateridae	Cardiophorini	sp.1		X	
Insecta	Coleoptera	Scydmaenidae		sp.7	X	X	P
Insecta	Diptera	Chironomidae		sp.	X		E
Insecta	Diptera	Culicidae	*Culex*	sp.	X		P
Insecta	Diptera	Psychodidae	*Sciopemyia*	*sordellii*	X	X	
Insecta	Heteroptera	Dipsocoroidea		sp.	X		E/P
Insecta	Heteroptera	Reduviidae	Reduviinae	sp.	1		P
Insecta	Homoptera	Cixiidae		jovem	X	X	P/A
Insecta	Hymenoptera	Formicidae	*Acromyrmex*	sp.1		X	
Insecta	Hymenoptera	Formicidae	*Hypoponera*	sp.1	X	X	P
Insecta	Hymenoptera	Formicidae	Myrmicinae	sp.1	X		P
Insecta	Hymenoptera	Formicidae	*Pachycondyla*	sp.1	X		P
Insecta	Hymenoptera	Formicidae	*Solenopsis*	sp.1	X		A
Insecta	Isoptera	Termitidae	*Nasutitermes*	sp.	X		P
Insecta	Lepidoptera	Gelechioidea		sp.	X		P
Insecta	Lepidoptera	Tineoidea		sp.		X	
Insecta	Orthoptera	Phalangopsidae	*Paraclodes*	sp.	1		E

Insecta	Orthoptera	Phalangopsidae	*Phalangopsis*	sp.	38		P/A
Insecta	Psocoptera	Psyllipsocidae		jovem	X	X	E
Insecta	Thysanura	Ateluridae		jovem	X		P
Insecta	Thysanura	Nicoletiidae		sp.1	X		E/A
Gastropoda	Pulmonata	Subulinidae	*Leptinaria*	sp.		X	
Gastropoda	Pulmonata	Systrophiidae	*Happia*	sp.		X	
Malacostraca	Isopoda	Armadillidae		sp.1	X	X	P
Malacostraca	Isopoda	Philosciidae		sp.		X	
Amphibia	Anura	Strabomantidae	*Pristimantis*	cf. *fenestratus*	1		P
Mammalia	Chiroptera	Emballonuridae	*Peropteryx*	sp.		X	
Mammalia	Chiroptera	Furipteridae	*Furipterus*	*horrens*	5		P
